# Evaluation of Postoperative Outcome and Incidence of Complications in Multisegment Le Fort I Osteotomies: A Case Series

**DOI:** 10.7759/cureus.39772

**Published:** 2023-05-31

**Authors:** V Manishaa, Sneha Pendem, Muthusekhar M R, Senthil Murugan Pandurangan

**Affiliations:** 1 Oral and Maxillofacial Surgery, Saveetha Dental College and Hospital, Chennai, IND

**Keywords:** necrosis, lefort, vascular compromise, orthognathic surgery, segmental lefort

## Abstract

Orthognathic surgeries are considered successful on the basis of postoperative stability, vascularity, and relapse rates. One among them is the multisegment Le Fort I osteotomy, which has often been disregarded due to vascular compromise. The complications associated with such an osteotomy are also primarily due to vascular ischemia. In the past, it was hypothesized that segmentation of the maxilla disrupted vascular supply to the osteotomized segments. However, the case series attempts to analyze the complications associated with a multisegment Le Fort I osteotomy and its incidence. This article documents four such cases that involve a Le Fort I osteotomy along with anterior segmentation. The patients experienced minimal or no postoperative complications. Thus, the case series exhibits that multisegment Le Fort I osteotomies can be successfully carried out without much complications and hence prove to be a safe treatment option in cases of increased advancement, setback, or combination.

## Introduction

Le Fort I osteotomy involves mobilization and repositioning of the maxilla in the superior, inferior, posterior (setback), or anterior (advancement). For any surgical procedure to be successful, the vascular supply to the postoperative sites must be adequate. In the case of an osteotomy procedure, the chances of vascular compromise due to damage to the blood vessels are common [1]. As a result, to maintain sufficient perfusion, meticulous planning with regard to incision and osteotomy must be carried out.

The common complications associated with Le Fort I osteotomy are devitalization of teeth, loss of teeth, formation of periodontal defects, necrosis of gingival tissues, avascular necrosis of entire dentoalveolar segments, and delayed union or nonunion [2]. Several studies have suggested that the interruption of the blood supply to the operative site is the cause of these complications [3]. Guidelines outlining the importance of preoperative planning, surgical procedures, and postoperative care have been included in such studies. 

In a study by Lanigan et al. [4], several cases of Le Fort I osteotomies with ischemia-related complications were studied. They formulated a list of surgical steps like improper flap designs, incisions, the excess elevation of the flaps, segmentalization, and transection of the descending palatine arteries as having the ability to decrease or compromise vascular supply to the maxillary tissues. It also advocated avoiding segmentation. However, later studies on rhesus monkeys have concluded otherwise [5]. They have studied revascularization patterns that enable surgeons to customize segmentation and osteotomy designs according to their requirements without hampering the blood supply. These studies have shown the possibility of segmentation and advancing the maxilla by up to 10 mm with adequate postoperative blood supply [6]. This case series documentation aims to attempt to highlight that multisegment Le Fort I osteotomies can be performed without causing any untoward damage to the vascular pedicle and with minimal complications.

## Case presentation

Four patients who reported to the Department of Oral and Maxillofacial Surgery and fulfilled the criteria for orthognathic surgery were included in this study. These patients were planned for a multisegment Le Fort I osteotomy due to the requirement for a higher magnitude of movement. One such case is discussed below.

**Figure 1:**
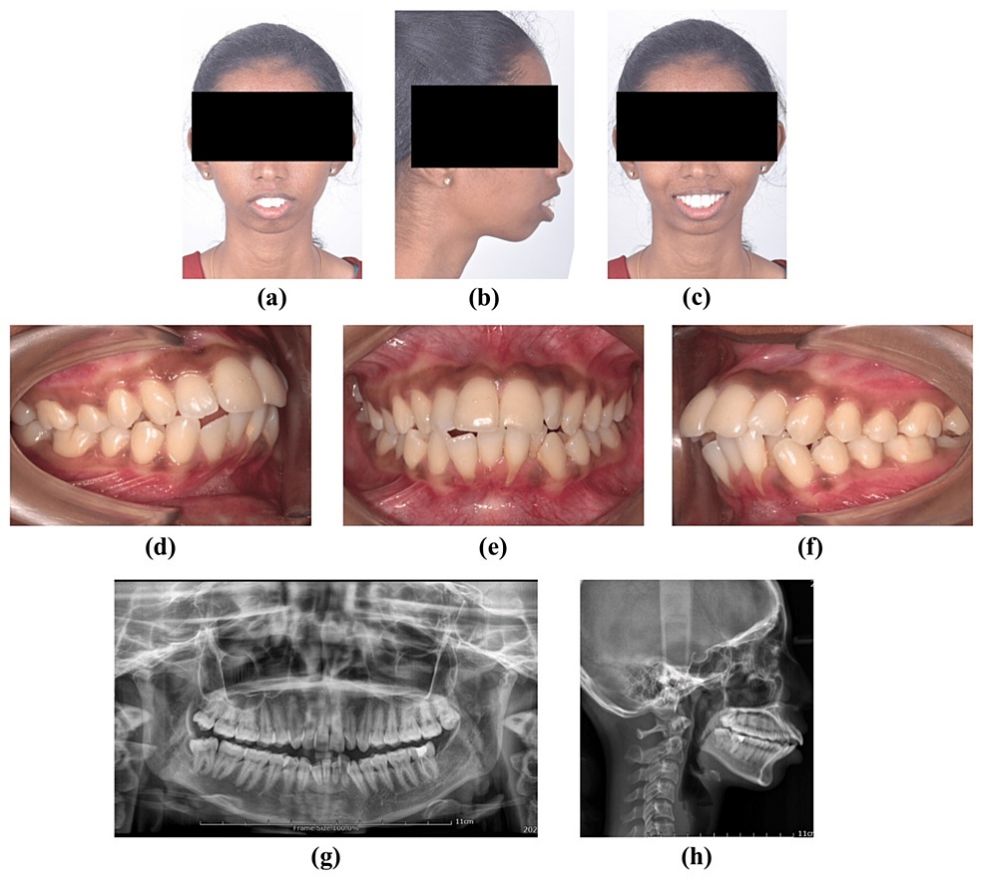
Preoperative records (a) Preoperative frontal at rest. (b) Preoperative profile at rest. (c) Preoperative frontal smile. (d) Preoperative occlusion on the right. (e) Preoperative occlusion frontal. (f) Preoperative occlusion on the left. (g) Preoperative orthopantamograph. (h) Preoperative lateral cephalography.

**Figure 2:**
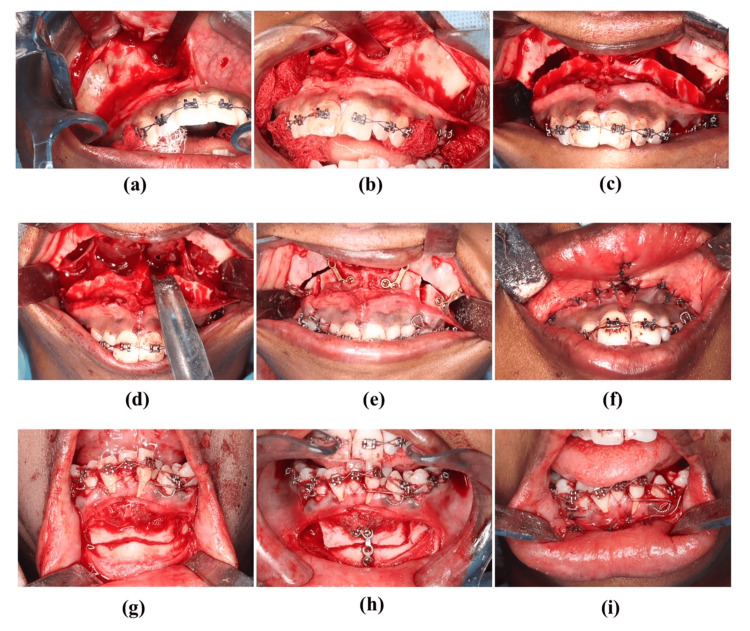
Intraoperative photographs (a) Exposure of the right maxilla. (b) Exposure of the left maxilla. (c) Le Fort I osteotomy. (d) Downward fracture of the segment. (e) Fixation of the multiple segments. (f) V-Y Closure of the maxillary incision. (g) Mandibular anterior subapical osteotomy. (h) Fixation. (i) Closure of the mandibular incision.

A 22-year-old female patient reported to the Department of Oral and Maxillofacial Surgery with complaints of forwardly placed upper teeth. Clinical examination revealed Angle’s Class I malocclusion with proclined upper and lower incisors. Extraoral examination revealed a convex profile, incompetent lips, and excessive incisors at rest. An intraoral examination revealed crowding in the upper and lower teeth (Figure 1). Pre-treatment radiological evaluation according to Steiner’s lateral cephalometric norms showed SNA angle = 88.2°, SNB = 74.2°, ANB = 7.3°. The first orthodontic approach was planned. The final diagnosis was skeletal Class II malocclusion. Le Fort I osteotomy setback by 4 mm and superior impaction by 5 mm with an anterior maxillary osteotomy was done under general anesthesia (Figure 2). Alar cinching and septoplasty were done to prevent nasal septum deviation.

**Figure 3:**
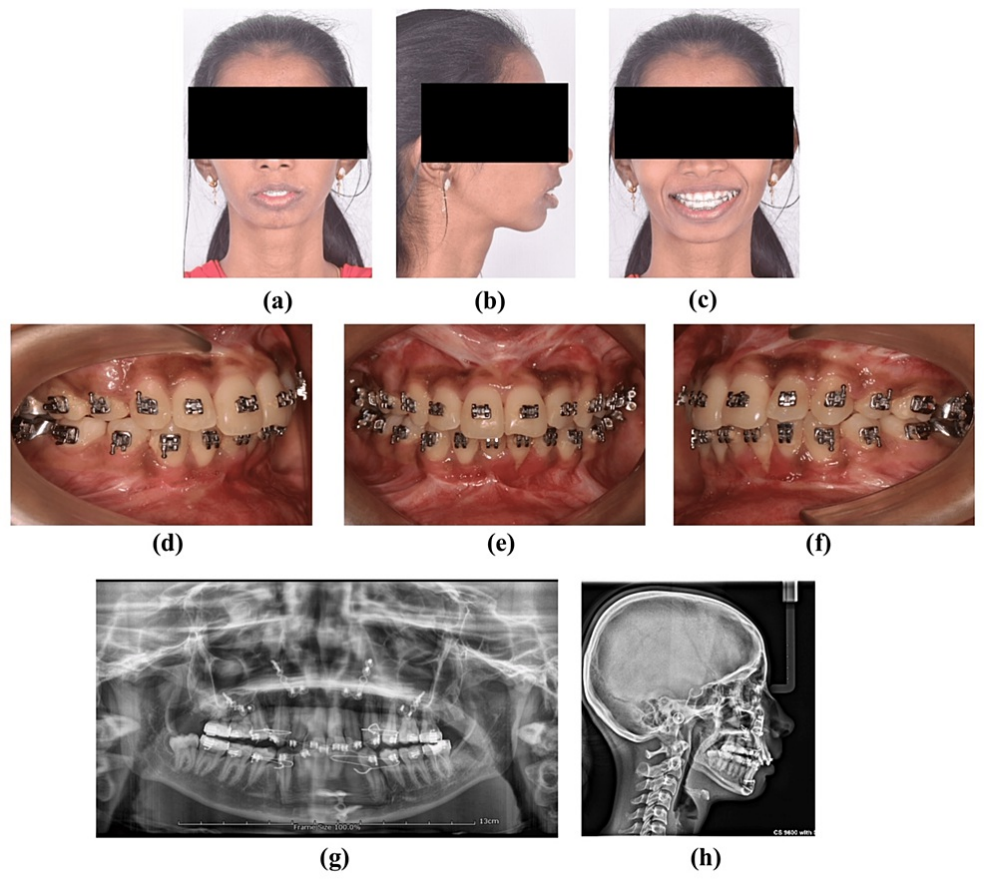
Postoperative records (a) Postoperative frontal at rest. (b) Postoperative profile at rest. (c) Postoperative frontal smile. (d) Postoperative occlusion on the right. (e) Postoperative occlusion frontal. (f) Postoperative occlusion on the left. (g) Postoperative orthopantamograph. (h) Postoperative lateral cephalograph

The patient was advised to follow up at the first and sixth weeks and three months postoperatively. The patient showed marked profile changes after the surgery (Figure 3). The patient was advised to undergo postoperative orthodontics for the correction of minor occlusal discrepancies for 8-10 months. 

A similar treatment plan and surgical protocol were followed for the other three patients. A multisegment Le Fort I osteotomy was done, and the patients were followed up to three months postoperatively. The results and complications encountered have been tabulated in Table 1. The incidence of complications in these reported cases has been tabulated in Table 2.

**Table 1:** Incidence of complications Description and incidence of complications occurring postoperatively in multisegment Le Fort I osteotomy

Complications	Incidence
Mobile teeth adjacent to osteotomy site	2
Loss of teeth adjacent to osteotomy site	0
Mobility of segment	0
Sloughed maxilla	0
Shaving of teeth adjacent to osteotomy	2
Tooth sensitivity	3
Massive hemorrhage	0
Postoperative infection	0
Flap dehiscence	0
Relapse	0
Non-vital teeth	1
Oro-antral fistula	0

**Table 2:** Case summary A summary of the cases included in the series

Case No.	Diagnosis	Treatment Done	Incidence of Complications
1	Skeletal Class II malocclusion	Le Fort I osteotomy setback by 6 mm and superior impaction by 5 mm with anterior maxillary osteotomy and mandibular anterior subapical setback by 4 mm were done.	Shaving of teeth adjacent to osteotomy, tooth sensitivity
2	Skeletal Class II malocclusion	Le Fort I osteotomy setback by 4 mm and superior impaction by 8 mm with an anterior maxillary osteotomy were done.	Mobile teeth adjacent to the osteotomy site
3	Skeletal Class II malocclusion	Le Fort I osteotomy setback by 4 mm and superior impaction by 5 mm with anterior maxillary osteotomy and mandibular anterior subapical setback by 5 mm were done.	Non-vital teeth, tooth sensitivity
4	Skeletal Class II malocclusion with posterior supraeruption of teeth.	Le Fort I osteotomy setback by 5 mm and superior impaction by 4 mm with posterior maxillary osteotomy and advancement genioplasty were done.	Mobile teeth adjacent to the osteotomy site, shaving of teeth adjacent to osteotomy, tooth sensitivity

## Discussion

Orthognathic surgeries were not initially done for aesthetic purposes. The maxillary osteotomy was first performed in the 1800s as an access osteotomy for the management of a nasopharyngeal tumor [7]. Wassmund was the first to document and publish his work on maxillary osteotomy to correct dentofacial deformity [8]. 

Le Fort I osteotomy is the most common maxillary osteotomy performed. The most common complications of osteotomy are due to vascular compromise. Earlier, several modifications in the techniques of separation and mobilization were made to maintain the vascular supply of the maxilla. Though the palatal incision provides maximum access to the osteotomy, it severely affects the blood supply. Hence, Kole, in 1959 [9], utilized a vestibular incision that was advantageous with good access and vascularity. A few years later, in 1965, Obwegeser introduced the standard wide incision involving the majority of the buccal vestibule to reposition the maxilla without tension [10]. 

Bell et al. conducted several studies on adult rhesus monkeys to understand the biological basis and vascular pattern of total, anterior, and posterior maxillary osteotomies individually [5,6,11]. The results of the animal studies revealed that all the aforementioned procedures were biologically sound. The intraosseous and soft tissue collateral circulation and the freely anastomosing gingival, palatal, floor of the nose, maxillary sinus, and periodontal plexus were adequate to provide vascular supply to the osteotomized segments, and the integrity of the incisive and greater palatine arteries was not significant. The authors concluded that osteotomies had to be made away from the tooth apices, and minimal elevation of the mucoperiosteal on either surface was sufficient to maintain intraosseous and intrapulpal circulation. Under such circumstances, segmental Le Fort I osteotomy can be carried out in any of the desired directions [11]. Another study by Nelson R. et al. on macaque monkeys to analyze blood flow with three different surgical approaches for anterior maxillary osteotomy also concluded that either a palatal, labial, or combined mucoperiosteal pedicle is adequate to provide vascularity to the segments after osteotomy, irrespective of the technique [12].

Quejada et al. in 1986 documented a newer technique utilizing bilateral minimal posterior horizontal and a midline vertical incision on the buccal aspect and subperiosteal tunneling to complete the buccal osteotomy and a palatal midline incision for the palatal osteotomy, respectively [13]. He then went on to perform a four-piece segmental maxillary osteotomy in five adult rhesus monkeys and evaluate the vascularity after sacrificing the animals on the 28th day. Their study results revealed that the soft tissue flap with minimal incisions provided adequate vascularity to the segments. Only transient ischemic changes and non-progressive marginal osteonecrosis were observed [14]. 

In 1990, Lanigan et al. conducted a questionnaire study among oral and maxillofacial surgeons and concluded that the risk of aseptic necrosis was increased in cases of multisegment Le Fort I osteotomies and in cases of previous history of cleft palatal repair [4]. This was more common in cases of superior impaction and transverse expansion. The authors advocate preserving the descending palatine arteries and minimal segmentation to avoid aseptic necrosis. On the contrary, Dodson et al. in 1997 compared the effects of ligation of the descending palatine artery during Le Fort I osteotomy on maxillary perfusion and concluded that there was no significant difference in ligating the artery, and blood flow through the osteotomized segments was similar when evaluated using laser Doppler flowmetry [15]. 

In 2010, a randomized controlled trial was conducted in 42 patients undergoing Le Fort I osteotomy to study the vitality of the maxillary anterior teeth [16]. All of the patients, except a minor 3.2%, required root canal therapy as the teeth were non-vital and had apical lesions. The study also adds that orthodontic therapy accentuates the sensitivity when excess forces are applied. Hence, pulp and tooth-related complications post-Le Fort I osteotomy are minimal, as highlighted in this study, but follow-up is required. A similar study on adult rhesus monkeys shows a significant effect of Le Fort I osteotomy on the pulp, with the majority of the axonal degeneration recovering within 24 weeks [17].

In cases of maxillary hypoplasia in patients with cleft lip and palate, the vascular supply of the maxilla is already compromised due to scarring post-palatal repair surgery. Hence, additional careful measures are required to maintain an adequate nutrient pedicle to ensure proper blood supply. Many authors have reported necrosis of the segments even when the palatal pedicle was intact. James RD et al. modified the Le Fort I, II, and III osteotomies with transection of the hard palate and performed them in postoperative cleft lip and palate patients to correct maxillary hypoplasia. They concluded that the modified technique and post-surgical craniomaxillary fixation aided in better viability and stability.

## Conclusions

This case series helps us to conclude that, in accordance with previous literature, segmentation of the maxilla does not compromise its vascular supply. However, to prevent any complication, several guidelines are to be followed that vary depending on the surgical technique employed, skills and ability of the surgeon, patient motivation and cooperation postoperatively, and operating time. These factors play a pivotal role in the incidence of complications pertaining to vascularity. A larger sample size and longer follow-up period are necessary to identify relapse and complications. 
